# Redox regulation in tumor cell epithelial–mesenchymal transition: molecular basis and therapeutic strategy

**DOI:** 10.1038/sigtrans.2017.36

**Published:** 2017-08-18

**Authors:** Jingwen Jiang, Kui Wang, Yan Chen, Haining Chen, Edouard C Nice, Canhua Huang

**Affiliations:** 1 State Key Laboratory of Biotherapy and Cancer Center, West China Hospital, Sichuan University, and Collaborative Innovation Center for Biotherapy, Chengdu, China; 2 Department of Gastrointestinal Surgery, State Key Laboratory of Biotherapy and Cancer Center, West China Hospital, Sichuan University, Collaborative Innovation Center for Biotherapy, Chengdu, China; 3 Department of Biochemistry and Molecular Biology, Monash University, Clayton, Victoria, Australia

## Abstract

Epithelial–mesenchymal transition (EMT) is recognized as a driving force of cancer cell metastasis and drug resistance, two leading causes of cancer recurrence and cancer-related death. It is, therefore, logical in cancer therapy to target the EMT switch to prevent such cancer metastasis and recurrence. Previous reports have indicated that growth factors (such as epidermal growth factor and fibroblast growth factor) and cytokines (such as the transforming growth factor beta (TGF-β) family) are major stimulators of EMT. However, the mechanisms underlying EMT initiation and progression remain unclear. Recently, emerging evidence has suggested that reactive oxygen species (ROS), important cellular secondary messengers involved in diverse biological events in cancer cells, play essential roles in the EMT process in cancer cells by regulating extracellular matrix (ECM) remodeling, cytoskeleton remodeling, cell–cell junctions, and cell mobility. Thus, targeting EMT by manipulating the intracellular redox status may hold promise for cancer therapy. Herein, we will address recent advances in redox biology involved in the EMT process in cancer cells, which will contribute to the development of novel therapeutic strategies by targeting redox-regulated EMT for cancer treatment.

## Introduction

In the early 1980s, Elizabeth Hay observed a phenotypic transition of epithelial cells to acquire mesenchymal characteristics.^1^ This differentiation process is now widely known as epithelial–mesenchymal transition (EMT), which is involved in embryonic development, wound healing and cancer progression.^2,3,4,5^ Epithelial and mesenchymal cells can be discriminated by distinct morphologic features: epithelial cells are laterally conjoined to form layers or polarized sheets, whereas mesenchymal cells rarely exhibit conjunctions with adjacent cells.^6^ Epithelial cells can be recognized by so-called epithelial markers, including claudins, E-cadherin, Crumbs3 and PALS1, all of which are critical for maintaining cell–cell junctions and cell polarity. By contrast, mesenchymal cells are characterized by key migration-promoting genes, such as N-cadherin, vimentin, α-smooth muscle actin (α-SMA) and fibroblast activation protein (FAP).^7^ The major changes of epithelial cells undergoing EMT include the following: (1) diminished cellular conjunctions (including adherens junctions, tight junctions, gap junctions and desmosomes); (2) decreased focal adhesion; (3) downregulated epithelial markers and upregulated mesenchymal markers; (4) increased cell mobility; (5) remodeled cytoskeleton; (6) and degraded ECM.^8,9^ Several transcription factors, such as Snail, Slug, Twist, ZEB1 and ZEB2, are responsible for repressing epithelial markers and upregulating genes associated with the mesenchymal phenotype.^10,11^ These transcription factors are tightly regulated by the nuclear factor-κB (NF-κB), hypoxia-inducible factor 1 (HIF-1) and transforming growth factor beta (TGF-β) signaling pathways. In addition, the transcription factor forkhead box class O (FoxO) can modulate ECM turnover and cell mobility by promoting the expression of matrix metalloproteinases (MMPs).^12^ Importantly, the subcellular localization of β-catenin is also critical for regulating the EMT process. On the cytomembrane, β-catenin tightly interacts with E-cadherin to maintain cell adhesion. When Wnt signaling is activated, β-catenin dissociates from E-cadherin, translocates to the nucleus and binds with TCF/LEF to activate the transcription of Snail, Twist and MMP-7.^13^ Although various transcription factors and modulators of EMT have been extensively studied, the precise mechanisms underlying EMT progression remain unclear. Importantly, a number of key EMT regulators were recently found to be redox-sensitive, enabling the elucidation of the molecular basis underlying the EMT process from a redox perspective.^14,15,16^

ROS are defined as oxygen-containing species that have highly reactive properties, and include free radicals such as hydroxyl free radicals (HO^•^), superoxide (O_2_^•−^) and non-radical molecules such as hydrogen peroxide (H_2_O_2_).^17,18^ ROS are important second messengers that regulate multifarious signaling pathways involved in cell proliferation, apoptosis, autophagy, migration, DNA damage, inflammation and drug resistance.^19,20,21^ Recently, the reversible and irreversible oxidative modifications of redox-sensitive proteins that possess free thiols (-SH) on cysteine residues, which are susceptible to ROS, have been found to play a crucial role in regulating signaling pathways. Several patterns of oxidative modifications have been reported, including sulfenylation (-SOH), sulfinylation (-SO_2_H), sulfonylation (-SO_3_H), S-glutathionylation (PrS-SG) and disulfide bonds (intramolecular, intermolecular and mixed types).^22^ Through these redox modifications, ROS can alter the biological functions of redox-sensitive proteins involved in ECM remodeling (for example, integrin, Hu antigen R), cytoskeleton remodeling (for example, actin, cofilin), cell–cell junctions (for example, NF-κB, HIF-1α, TGF-β) and cell mobility (for example, Src, FAK, PTEN), thereby regulating EMT initiation and cancer cell progression.^9^

In this review, we will highlight recent progress in understanding the molecular basis of redox-regulated EMT in cancer cells and discuss the opportunities and challenges for targeting redox-regulated EMT as a potential therapeutic strategy for cancer.

## Redox regulation of ECM remodeling

During EMT, ECM proteins undergo degradation to confer cells with invasive potential. ECM stabilization is closely correlated with the expression of MMPs. The urokinase plasminogen activator (uPA) pathway has been reported to participate in ECM turnover and could be regulated by ROS. Integrins are believed to be important transmembrane proteins that link intracellular signaling mediators and ECM proteins. When EMT is initiated, the original integrins will be downregulated, and other types of integrin will be expressed, leading to new interactions with different ECM components to promote cell invasion.^23^ Recent studies have suggested that ECM remodeling could be mediated by redox regulation. Some integrins function as redox sensors; thus, this information may reveal a new perspective for uncovering the mask of ECM remodeling and integrin arrangement.

### Integrin arrangement

**Figure 1:**
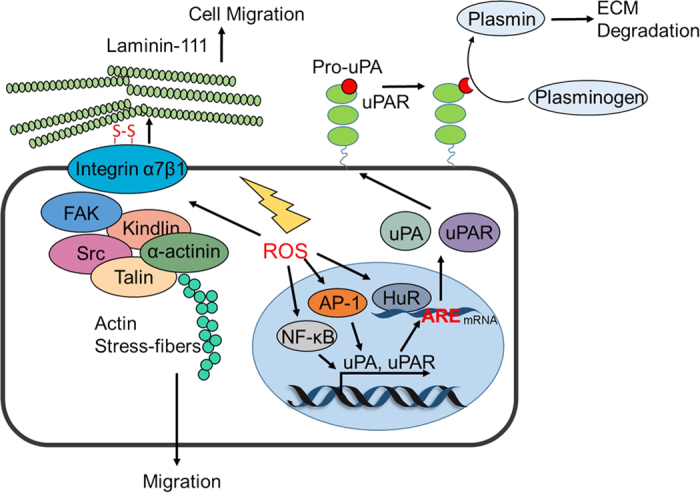
Redox regulation of ECM remodeling. Integrins can undergo oxidative modification by ROS during the initiation of EMT. Oxidized integrin α7β1 binds to laminin-111 and activates its downstream proteins, including FAK and Src. The uPA system is also tightly regulated by ROS. ROS promote the transcription of uPA and uPAR by activating the AP-1 and NF-κB signaling pathways. Moreover, ROS can stabilize uPA and uPAR mRNA by enhancing the binding of HuR with ARE^uPA^, leading to overexpression of uPA and uPAR and the subsequent activation of plasmin. ECM, extracellular matrix; EMT, epithelial–mesenchymal transition; FAK, focal adhesion kinase; uPA, urokinase plasminogen activator; ROS, reactive oxygen species; uPAR, urokinase plasminogen activator receptor; AP-1, activator protein-1; NF-κB, nuclear factor-κB; HuR, Hu antigen R; ARE, AU-rich elements.

Integrins are cell surface adhesion molecules that link the ECM to the intracellular actin cytoskeleton. They are heterodimeric receptors consisting of 18 α-subunits and 8 β-subunits, which can assemble into at least 20 different integrins. These integrins differentially act to regulate cellular processes by binding to selective extracellular substrates.^24^ The extracellular domain of integrins can bind ECM proteins such as fibronectin, laminin and collagen. This binding causes the integrins to undergo conformational changes, exposing their cytoplasmic tail, which leads to linkage with the actin cytoskeleton.^25^ Moreover, integrin engagement can activate Src and FAK, both of which are involved in coordinating the reorganization of the actin cytoskeleton.^26^ It has been confirmed that integrin α7β1 is a redox sensor that can be oxidized and activated by ROS (generated by NADPH oxidase 4, NOX4). Treatment with diphenylene iodonium chloride (DPI, an inhibitor of NOX) can rescue the attachment of rat aortic smooth muscle cells to laminin-111.^27^ Two sites on integrin α7 have been identified to be modulated by H_2_O_2_, one being located in the genu region and another in the calf 2 domain. Within the calf 2 domain, Cys862 can form different disulfide bridge-stabilized conformations by cross-linking with Cys916, Cys923, or Cys928. In the genu region, 2 cysteine residues (Cys604 and Cys610) can simultaneously undergo oxidized modifications by ROS, contributing to the conformational switch, finally leading to the activation of integrin α7β1 and its binding with laminin-111 (Figure 1).

### The uPA pathway

Urokinase plasminogen activator (uPA) is an extracellular serine protease that can be activated by binding to its specific receptor uPAR. Activated uPA mediates the cleavage of plasminogen to form plasmin, which is required for ECM degradation and MMP activation. High uPAR expression is associated with poor prognosis of patients with multiple types of cancer, suggesting a positive correlation between upregulated uPAR and tumor malignancy.^28^ It has been reported that ROS can induce the transcription of both uPA and uPAR by regulating activator protein-1 (AP-1) and NF-κB.^29^ Moreover, the mRNA-stabilizing factor Hu antigen R (HuR), which binds to the instability-determining AU-rich elements (ARE) of uPA and uPAR, can also be regulated by ROS.^30^ H_2_O_2_ treatment promotes the phosphorylation of mitogen-activated protein kinase-activated protein (MAPKAP) kinase 2 (MK2) and enhances the binding of HuR with ARE^uPA^, thus increasing the expression of uPA and uPAR (Figure 1). The intrinsic links between HuR and oxidative stress were further strengthened by the finding that HuR silencing accounts for increased ROS levels in triple-negative breast cancer (TNBC).^31^ Excessive ROS caused by HuR knockdown led to the radiosensitization of tumor cells, providing a potential strategy for the treatment of advanced breast cancer. However, the actual mechanisms underlying how ROS mediates the binding of HuR and ARE^uPA^ requires further investigation.

## Redox regulation of cytoskeleton remodeling

Cytoskeleton remodeling is important for regulating cell elongation and contractility.^32^ Although cofilin and moesin have been reported to regulate dynamic actin reorganization, the mechanism underlying the subtle control of actin dynamics remains unclear. Recently, increasing evidence has suggested that ROS play important roles in the regulation of dynamic actin polymerization.

**Figure 2:**
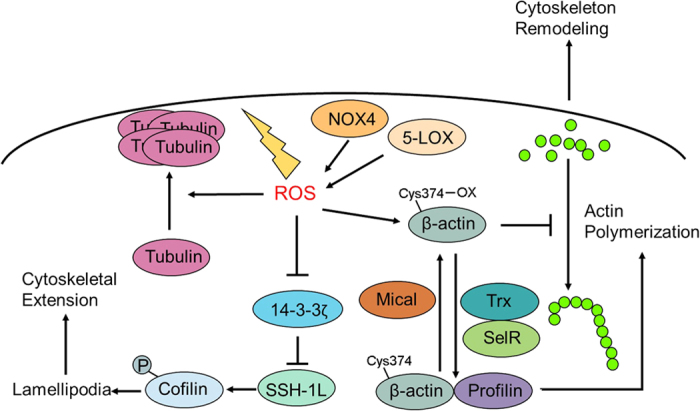
Redox regulation of cytoskeleton remodeling. Cys374 of β-actin can be oxidized in response to oxidative stress, leading to decreased actin polymerization and enhanced cytoskeleton remodeling. Mical promotes the oxidation of β-actin, whereas Trx and SelR mediate the reduction of β-actin. In addition, tubulin can also be regulated by ROS. ROS promote the formation of tubulin tetramers, leading to cytoskeletal injury. Moreover, ROS can also affect the formation of lamellipodia by inhibiting the activity of 14-3-3ζ. Once oxidized, the inhibitory effect of 14-3-3ζ on SSH-1L will be alleviated, ultimately leading to the formation of the cofilin-actin rod and lamellipodia. Trx, thioredoxin; ROS, reactive oxygen species; SSH-1L, Slingshot-1L; NOX4, NADPH oxidase 4; 5-LOX, 5-lipoxygenase.

The cytoskeleton is composed of the microtubule network, intermediate filaments and the actin cytoskeleton, and it plays important roles in controlling cell shape. Specifically, actin cytoskeleton remodeling acts as a driving force for cell migration due to the dynamic alteration of cellular protrusions that occurs.^33^ The dynamic actin network is controlled by Rac, an upstream regulator of NOX, implying that ROS may be involved in actin cytoskeleton regulation. Recent studies have shown that Rac-mediated actin remodeling is attributed to increased O_2_^•−^ levels. Deletion of O_2_^•−^ using DPI or MnTMPyP reduced actin elongation and endothelial cell migration.^34^ Notably, actin can be directly oxidized by ROS. Cys374 in the C-terminal region of β-actin can be oxidized in both the G-actin (monomeric) and F-actin (polymerized) forms, with the oxidized form of actin slowing actin polymerization.^35^ Moreover, oxidized β-actin has been reported to promote actin depolymerization, partially due to decreased affinity with profilin. Another study found that β-actin could form mixed disulfides with glutathione. The reversion of actin glutathionylation (for example, by inhibiting 5-lipoxygenase) contributes to the inhibition of actinomyosin disassembly.^36^ ROS accumulation induced by Mical enzymes is also linked to the remodeling of F-actin via oxidative modifications of cytoskeletal and nuclear actin.^37,38^ Thioredoxin (Trx) and SelR enzymes are now known to reverse the oxidative modification of actin. The Mical enzymes together with SelR reductases orchestrate the assembly and disassembly of actin filaments through a redox regulatory mechanism (Figure 2).^39^ Similar to actin, tubulin is another critical component of the cytoskeleton that can be modulated by oxidative stress.^40^ In the presence of H_2_O_2_ or HOCl, tubulin can form tetramers by oxidative modification.^41^ In addition, treatment with paraquat, a ROS inducer, results in cytoskeletal injury by diminishing tubulin and microtubules.^42^ These studies indicate that oxidative stress monitors cytoskeleton remodeling via the redox regulation of both actin and tubulin.

Lamellipodia and filopodia are actin-rich membrane projections that act as sensory extensions of the cytoskeleton. Lamellipodia are flat protrusions that are distributed with highly branched actin filaments to form the leading edges of migrating cells.^43^ The actin-binding protein cofilin is a major regulator of actin reorganization and lamellipodia formation. Cofilin activation can be modulated by its phosphorylation status, which is controlled by Slingshot (SSH) and Slingshot-1L (SSH-1L). Notably, ROS can modulate the activity of SSH-1L by mediating the association of SSH-1L and its negative regulator 14-3-3ζ. Once oxidized by ROS, 14-3-3ζ can reverse the inhibition of SSH-1L, ultimately leading to the formation of a cofilin-actin rod (Figure 2).^44^ Consistently, upon exposure to ROS, retrograde F-actin flow is dramatically potentiated in lamellipodium.^45^ The involvement of ROS in regulating actin reorganization was further substantiated by investigating the influence of oxidized actin on the actomyosin complex. Oxidized actin could promote the contractility of actomyosin by inducing the disassembly of actin and myosin, leading to the formation of stress fibers and the promotion of cell spreading. ROS elimination with GSH or overexpression of the β-actin C374A mutant dramatically inhibited the disassembly of actomyosin.^46^ Further investigations are required to clarify the interplay between ROS generation and actin cytoskeleton remodeling to understand in depth how EMT and cell migration occurs.

## Redox regulation of cell–cell junctions

Cell–cell junctions are essential for maintaining epithelial integrity.^47^ EMT initiation causes the diminishment of cell junctions and the deconstruction of epithelia-formed permeability barriers. Loss of tight junctions is commonly accompanied by decreased expression of occludin and claudin, whereas E-cadherin degradation is pivotal for the dissolution of adherens junctions.^48,49^ Moreover, during the destabilization of gap junctions, the expression of connexin is dramatically decreased, leading to the loss of cell–cell communication. These junction proteins are repressed during EMT by transcription factors such as Snail, Slug, Twist and ZEB. It is well-established that these key EMT-inducing transcription factors are regulated by the convergence of signaling pathways, including the NF-κB, HIF-1 and TGF-β pathways.^50,51,52^ These signaling pathways modulate not only EMT-inducing transcription factors but also key regulators involved in cell mobility and cytoskeleton remodeling. Below, we will discuss the role of redox regulation in these signaling pathways and highlight the importance of ROS in EMT initiation.

### NF-κB

**Figure 3:**
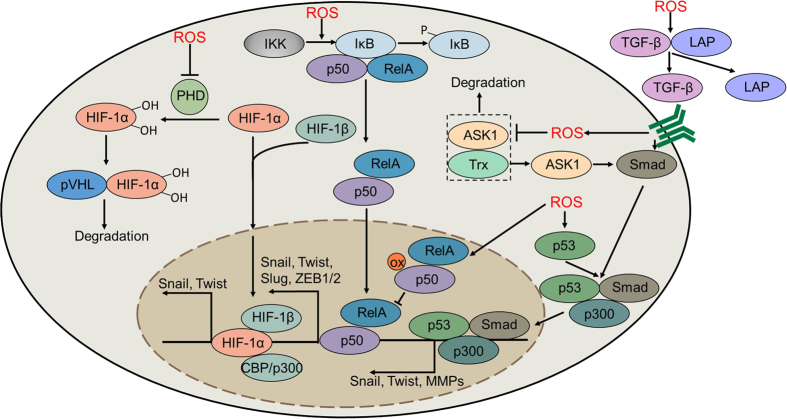
Redox regulation of cell–cell junctions. HIF-1α can be hydroxylated by PHD and subsequently undergoes degradation by interacting with pVHL. ROS inhibit the activity of PHD to stabilize HIF-1α, thus inducing the transcription of Snail and Twist. Moreover, ROS promote the IKK-mediated degradation of IκB and induce the nuclear translocation of NF-κB, leading to the transcriptional activation of Snail, Slug, Twist and ZEB1/2. However, ROS also inhibit the NF-κB signaling pathway by oxidizing p50 in the nucleus. ROS can also activate the TGF-β signaling pathway by enhancing the dissociation of LAP from TGF-β. Furthermore, ROS promote ASK1 activation by inhibiting the association of ASK1 and Trx, leading to the activation of Smad. In addition, p53 can be phosphorylated in response to oxidative stress, leading to the formation of p53/Smad/p300 complex, which initiates the transcription of Snail, Twist and MMPs. HIF-1α, hypoxia-inducible factor 1α; PHD, prolyl hydroxylase; pVHL, Von Hippel-Lindau protein; ROS, reactive oxygen species; IKK, IκB kinase; NF-κB, nuclear factor-κB; ZEB1/2, zinc finger E-box binding homeobox 1/2; TGF-β, transforming growth factor β; Trx, thioredoxin; ASK1, apoptosis signal-regulating kinase 1; LAP, latency-associated protein; MMPs, matrix metalloproteinases.

The NF-κB transcription factor family consists of 5 members: p50, p52, p65 (RelA), RelB and c-Rel. All members have a Rel homology domain (RHD), which is required for dimerization and DNA binding.^53,54^ Normally, NF-κB is a hetero/homodimer that can be inactivated by inhibitors of kappa B (IκB) in the cytoplasm. In the presence of certain cellular stimuli, NF-κB signaling is activated by the IκB kinase (IKK) complex-mediated Ser32/36 phosphorylation of IκB.^55^ Activation of NF-κB signaling is strongly associated with the EMT process by promoting the expression of Twist1, Snail1, Slug and ZEB1/2, which contributes to the disruption of cell–cell junctions.^56^ Specifically, NF-κB activation also induces the transcription of vimentin and MMPs (such as MMP-2, MMP-9) to maintain the mesenchymal phenotype and promote tumor cell migration. Moreover, NF-κB is required for the stimulation of COP9 signalosome 2 (CSN2) to suppress the ubiquitination-mediated degradation of Snail, thereby resulting in cancer cell metastasis.^3^ Notably, it has been reported that oxidative stress plays a vital role in regulating NF-κB signaling (Figure 3). For instance, increased ROS levels can activate NF-κB signaling and induce EMT-related morphological changes, whereas administration of the antioxidant N-acetylcysteine (NAC) or the NF-κB-specific inhibitor DHMEQ significantly reverses ROS-induced EMT.^57,58,59^ Furthermore, ROS confer pancreatic cancer cells with invasive ability by activating NF-κB signaling. Catalase treatment reverses EMT initiation, indicating that H_2_O_2_ may play an important role in NF-κB-mediated EMT.^60^ In addition, TNFR (tumor necrosis factor receptor) activation induces ROS accumulation, leading to the phosphorylation and subsequent degradation of IκB.^61^ In the cytoplasm, NF-κB essential modulator (NEMO, also known as IKKγ) is activated by forming an intermolecular disulfide bond between Cys54 and Cys347 in response to oxidative stress.^62^ Even more perplexing, accumulating evidence suggests that ROS may inhibit NF-κB signaling. Notably, Cys62 of p50 can undergo glutathionylation in the nucleus, which inhibits its DNA-binding ability; however, this process can be reversed by Trx. Moreover, Kelch-like ECH-associated protein 1 (KEAP1) has been reported to inhibit NF-κB signaling via the H_2_O_2_-mediated degradation of IKKβ.^63^ These observations imply that ROS in diverse cellular compartments may lead to disparate consequences in redox-regulated NF-κB signaling.

### HIF-1

In addition to NF-κB signaling, HIF-1 can also promote cancer cell EMT by activating EMT-inducing transcription factors, such as Twist, Snail and ZEB1.^64,65^ Recent studies have demonstrated that ROS can suppress E-cadherin expression via the HIF-1-mediated overexpression of LOX in ovarian carcinoma cells.^66^ Furthermore, ROS have been reported to initiate EMT via the Y654 phosphorylation of β-catenin and subsequent activation of HIF-1 signaling.^67^ In addition, miR373 has been reported to induce EMT in breast cancer through the ROS-mediated activation of HIF-1α.^68^ HIF-1 consists of two subunits: the oxygen-sensitive HIF-1α subunit and the constitutively expressed HIF-1β subunit. The COOH-terminal (C-TAD) and NH_2_-terminal (N-TAD) domains are required for the transcriptional activity of HIF-1α. Under hypoxia, HIF-1α and HIF-1β form a heterodimer, which translocates into the nucleus and binds to hypoxia-response element (HRE).^55^ Under normoxic conditions, HIF-1α is hydroxylated on its proline residues and undergoes degradation. This process is mediated by prolyl hydroxylase (PHD), the activation of which requires O_2_, 2-oxoglutarate (2-OG), and ferrous iron (Fe^2+^). Under hypoxic conditions, Fe^2+^ is oxidized to Fe^3+^ by ROS accumulation. Concomitantly, the hydroxylation activity of PHD is inhibited, resulting in HIF-1α stabilization and subsequent activation of the HIF-1 pathway (Figure 3).^69^ Previous studies have suggested that PHD is not only an oxygen sensor during HIF-1α degradation but can also sense free cysteine residues. L-cysteine can activate PHD, and the oxidation of cysteine residues within its catalytic domain (Cys208, Cys266, Cys302 and Cys323/326) leads to the inactivation of PHD, indicating that free cysteine residues protect PHD from auto-oxidation.^70^ The von Hippel-Lindau (VHL) tumor suppressor, which targets HIF-1α for oxygen-induced proteolysis, has also been reported to be activated by ROS.^71^ Recently, Axl, a member of the receptor tyrosine kinase (RTK) family, has been found to be a direct target of HIF during EMT progression.^72^ Genetic or pharmacological inactivation of Axl leads to reversal of the invasive phenotype in clear cell renal cell carcinoma. Intriguingly, a positive feedback loop exists between Axl and ROS. Axl can enhance the accumulation of ROS by activating Rac1. Accordingly, H_2_O_2_ treatment results in an intensive phosphorylation of Axl and enhances cell migration by activating the PI3K/Akt cascade.^73^ However, the regulatory mechanism underlying ROS-mediated Axl activation remains elusive.

### TGF-β

TGF-β is an important profibrogenic cytokine that regulates cell proliferation and cell adhesion and plays a predominant role in regulating tumor cell EMT.^74^ TGF-β can suppress E-cadherin expression by activating Snail, leading to decreased adherens junctions.^75^ It has been proved that the activation of TGF-β signaling can trigger EMT by decreasing fibronectin levels, whereas interfering with ROS by exogenously expressing mitochondrial thioredoxin (TXN2) reverses TGF-β-induced EMT.^76^ Once activated, TGF-β can bind with type II receptor (TGFβR-II) and subsequently activate type I receptor (TGFβR-I), resulting in the phosphorylation of Smad2 and Smad3. Phosphorylated Smad2 and Smad3 then interact with Smad4 (Co-Smad) and translocate into the nucleus to initiate the transcription of target genes. ROS have been reported to be critical signaling intermediaries in the manipulation of TGF-β signaling.^77^ For example, ROS can stimulate the phosphorylation of p53 on Ser15, leading to the formation of a p53/SMAD/p300 complex that is responsible for the transcriptional activation of TGF-β.^78^ Moreover, H_2_O_2_ produced by mitochondria or NOX can elevate the mRNA and protein expression of TGF-β and activate TGF-β signaling.^79,80^ Latent TGF-β activation, a process during which secreted TGF-β is released from latency-associated protein (LAP), is necessary for the interaction between TGF-β and its receptors.^77^ Notably, it has been reported that LAP is sensitive to ROS. Oxidized LAP loses its binding capacity for TGF-β, leading to the activation of TGF-β signaling (Figure 3).^81^ In addition, recent studies have suggested that activation of the TGF-β/SMAD signaling pathway also requires ROS production. For instance, apoptosis signal-regulating kinase 1 (ASK1) can activate the TGF-β pathway in response to ROS.^82^ ASK1 is a MAPKK kinase (MAPKKK) that activates the p38 MAPK pathway by phosphorylating MKKs.^83^ Normally, Trx binds to ASK1, leading to the ubiquitination-mediated degradation of ASK1.^84^ Under oxidative stress, ASK1 undergoes multimerization by forming a disulfide bond, thereby disrupting its ability to bind Trx.^85,86,87^ These studies suggest a potential regulatory mechanism of ASK1 in TGF-β activation. Intriguingly, TGF-β can promote ROS production via the inhibition of mitochondria complex IV and the upregulation of NOX4.^88^ Moreover, TGF-β can regulate redox balance by directly disturbing the antioxidant system. Specifically, TGF-β can promote the deletion of GSH, a major intracellular reductant, leading to imbalanced intracellular redox homeostasis. Thus, redox-regulated TGF-β signaling orchestrates a positive feedback loop to enable EMT progression.

## Redox regulation of cell mobility

EMT is characterized by increased formation of actin stress fibers and actin rearrangement, which contributes to cell directional motility. Rho GTPases are involved in actin rearrangement and can be regulated by the focal adhesion kinase (FAK), Src and PI3K/Akt signaling pathways.^89^ Among the Rho GTPases, RhoA promotes the formation of actin stress fibers, and Rho-associated kinase (ROCK) induces actin polymerization by cooperating with formin diaphanous 1 (DIA1). Furthermore, CDC42 and Rac1 contribute to the formation of lamellipodia and filopodia.^90^ Next, we will highlight the redox regulation of key proteins involved in altering cell mobility in an attempt to outline the interconnection between redox balance and cell mobility alteration.

### FAK

**Figure 4:**
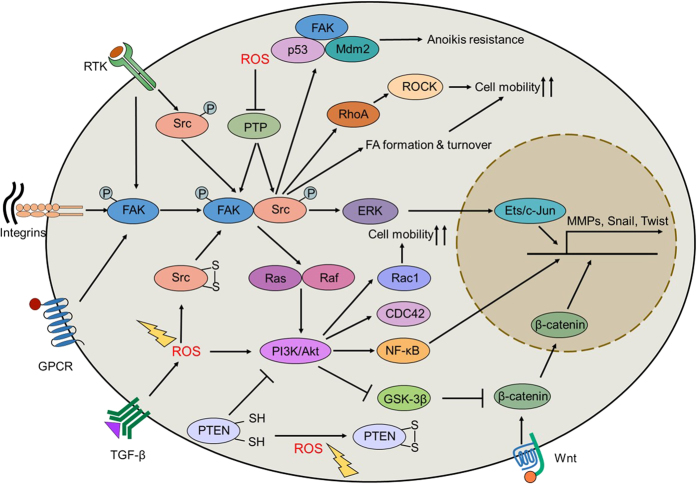
Redox regulation of cell mobility. ROS activate Src by inducing the formation of disulfide bonds. Activated Src binds with FAK to promote RhoA/ROCK signaling and FA formation. The Src-FAK complex also activates ERK and promotes the transcription of MMPs, Snail and Twist. Furthermore, FAK confers tumor cells with anoikis resistance by inducing Mdm2-mediated p53 degradation. In addition, PTP can also undergo oxidation and inactivation in response to oxidative stress, leading to inhibition of the FAK-Src complex. ROS inactivate PTEN by oxidative modification and activate the PI3K/Akt signaling pathway, leading to the activation of both Rac1/CDC42 and the NF-κB signaling pathways, which enhances cancer cell mobility. Moreover, the PI3K/Akt axis can inhibit GSK-3β activity and promote the nuclear-translocation of β-catenin, resulting in the transcription of Snail, Twist and MMPs. ROS, reactive oxygen species; FAK, focal adhesion kinase; ROCK, Rho-associated kinase; FA, focal adhesion; ERK, extracellular signal-regulated kinases; MMPs, matrix metalloproteinases; PTP, phosphotyrosine-phosphatase; PTEN, phosphatase and tensin homolog; PI3K, phosphoinositide 3-Kinase; CDC42, cell division cycle 42; NF-κB, nuclear factor-κB; GSK-3β, glycogen synthase kinase-3β; MMPs, matrix metalloproteinases.

FAK, a ubiquitously expressed non-receptor tyrosine kinase, is an important signal transduction mediator involved in cell spreading and migration through kinase-dependent or independent mechanisms.^91,92^ It has been reported that FAK can control cell mobility by recruiting talin to nascent adhesions.^93^ When cells associate with the ECM, the integrin receptor clusters, which induces FAK auto-phosphorylation at Y397, contributing to the formation of the activated FAK-Src complex.^94^ Following activation of FAK, the RhoA/ROCK pathway is activated, leading to the formation of focal adhesion (FA) and actin stress fibers. In addition, previous reports have revealed that the FAK-p130Cas complex can mediate matrix degradation by recruiting MT1-MMP to focal adhesions.^95^ Furthermore, FAK activation confers tumor cells with anoikis resistance by interacting with Mdm2 and promoting the subsequent proteasomal degradation of p53.^96^ Recent studies have suggested that ROS can regulate cell mobility by modulating FAK activation. Under oxidative stress, FAK shows decreased phosphorylation through the 4-hydroxy-2-nonenal (4-HNE)-dependent pathway. Antioxidants such as NAC and MPG attenuate the 4-HNE-mediated redistribution of FA and the formation of actin stress fibers.^97^ By contrast, ROS produced by NOXs increase FAK^Y397^ phosphorylation by inhibiting phosphotyrosine-phosphatase (PTP), resulting in focal adhesion stabilization and actin polymerization (Figure 4).^98^ However, oxidative modifications on specific cysteine residues of FAK are poorly documented, and further studies are required.

### Src

C-Src, a member of the Src tyrosine kinase (SFK) family, plays crucial roles in regulating cell survival, proliferation and migration.^99^ Src is overexpressed and/or hyper-activated in various human tumors due to the dysregulation of growth factor signaling pathways (for example, EGFR, VEGFR and FGFR) and integrin engagement. Activation of Src can enhance cell movement by promoting focal adhesion turnover. Src can also promote the detachment of tumor cells from the primary tumor by downregulating E-cadherin and upregulating MMPs. Furthermore, Src also disrupts tumor cell adhesion by cooperating with MAPK and ROCK to stimulate the peripheral accumulation of phospho-myosin, thus maintaining the mesenchymal phenotype of tumor cells.^26,100^ In addition, Src has been reported to phosphorylate cadherin adhesion components (such as p120-catenin) to decrease cell–cell adhesion.^101^ The activity of Src is dependent on the phosphorylation status of two regulatory tyrosine residues: Tyr416 and Tyr527. Tyr416 phosphorylation contributes to Src activation, whereas Tyr527 phosphorylation leads to Src inactivation.^14^ The phosphorylation of Tyr527 is regulated by C-terminal Src kinase (Csk) and Csk homology kinase (Chk), whereas dephosphorylation of Tyr527 is induced by PTPs, such as SH2-containing protein tyrosine phosphatase 1/2 (SHP-1 and SHP-2), PEST domain-enriched tyrosine phosphatase (PEP) and low-molecular-weight protein tyrosine phosphatase (LMW-PTP). In addition to the phosphorylation/dephosphorylation regulation patterns of Src, the activity of Src can also be monitored by intracellular ROS. Src possesses 5 conserved cysteine residues (Cys238, 245, 400, 487 and 498), of which two (Cys245 and Cys487) are responsible for its oxidative activation in response to ROS. Cys245 (in the SH2 domain) and Cys487 (in the kinase domain) can form an intramolecular disulfide bond to reinforce an active conformation (Figure 4).^102^ Recent studies have shown that tumor suppressor B-cell translocation gene 2 (BTG2) can inhibit Src-FAK signaling by decreasing the mitochondria-derived ROS levels in prostate cancer cells.^103^ In addition, the obliteration of NOX-generated ROS can also inactivate Src, which consequently influences cell mobility by restricting the phosphorylation and localization of Ezrin.^104^ Previous studies have suggested that a high ratio of O_2_^•−^ to H_2_O_2_ leads to the oxidation-mediated activation of Src, thus conferring anoikis resistance by activating the PI3K/PKBα and ERK pro-survival pathways.^105^ Reversible oxidation has emerged as an important mechanism for regulating the activity of PTPs, including SHP-1, SHP-2 and LMW-PTP.^106,107,108,109^ The oxidative modification of cysteine residues in the catalytic domain of PTP leads to the inhibition of PTP, thereby enhancing Src phosphorylation.^110,111,112^ Previous studies have indicated that Cys12 and Cys17 can form a disulfide bond to inactivate LMW-PTP under oxidative stress, which can be rescued by treatment with reductants.^113^ Therefore, the use of reductants may be a promising approach for the prevention of cancer metastasis by inhibiting Src activation.

### PI3K/Akt

The PI3K/Akt axis can facilitate protein synthesis and promote EMT by activating the NF-κB pathway.^114^ In addition, PI3K plays a critical role in regulating cell mobility by recruiting Rac1 and CDC42 to the leading edges of migrating cells.^115^ Furthermore, activation of the PI3K/Akt pathway is closely linked with GSK-3β inhibition, which endows the stabilization of β-catenin to activate the transcription of Slug and vimentin.^116^ Accumulating evidence has indicated that the PI3K/Akt pathway can be activated by ROS.^117^ For instance, the activity of phosphatase and tensin homolog (PTEN), a well-known negative regulator of PI3K/Akt signaling, is directly repressed by ROS. The reversible inactivation of PTEN by ROS is due to disulfide bond formation between Cys124 and Cys71 at the catalytic site, and Trx is required for deoxidation and restoration of PTEN activity. This direct oxidation results in the inhibition of PTEN activity, thus contributing to the activation of the PI3K/Akt cascade (Figure 4).^118,119^ The PI3K inhibitor wortmannin can impede ROS production by restricting the translocation of NOX subunits, suggesting the PI3K/Akt pathway may play a role in ROS production.^120^ However, the manner in which the PI3K/Akt pathway regulates ROS production is not fully understood, and further investigations are needed to reveal the redox-sensitive proteins involved in this regulatory pathway.

## Targeting redox-regulated EMT for cancer therapy

Tumor metastasis and drug resistance are two major obstacles in cancer therapy. Recent studies have shown that EMT acts as a critical regulator for not only driving tumor metastasis but also for modulating drug resistance. For example, the differentially expressed genes between paired erlotinib-resistant and erlotinib-sensitive pancreatic cancer cells were analyzed using gene expression profiling. The results demonstrated that the expression of a set of genes implicated in EMT was altered. Further studies have shown that ZEB1 silencing enhanced the therapeutic effect of erlotinib in resistant cancer cells.^121^ Moreover, oxaliplatin-resistant colorectal cancer cells exhibit a similar phenotype to EMT.^122^ Consistent with these observations, suppression of EMT enhanced the sensitivity of pancreatic cancer cells to gemcitabine.^123^ 5-Fluorouracil is a first-line therapeutic agent for various types of cancer. However, extended exposure to 5-fluorouracil leads to chemoresistance. Interestingly, tumor specimens from patients who have undergone more than one week of chemotherapy (combination of uracil, tegafur and 5-fluorouracil) prior to surgery displayed elevated expression levels of mesenchymal markers.^122^ Consistently, 5-fluorouracil-resistant pancreatic adenocarcinoma cells showed significantly upregulated mesenchymal markers and enhanced invasive potential. In addition, L1CAM, a chemoresistant and invasive phenotype-associated protein, was found to be dramatically upregulated in chemoresistant pancreatic cancer cells. Further investigation showed that Slug is responsible for the upregulation of L1CAM.^124^ Moreover, it has been increasingly recognized that antiandrogen treatment can retard drug resistance in prostate cancer by reversing the EMT process.^125^ Collectively, these findings highlight the important role of EMT in regulating drug resistance and suggest potential combined therapeutic strategies for the treatment of drug-resistant cancers.^126^

Anoikis, originally defined by Frisch and his colleagues, is a unique version of apoptotic cell death due to ECM detachment.^127,128^ During anoikis, both mitochondrial and death receptor-mediated cell death pathways are activated. Once detached from adjacent cells or the ECM, cells likely undergo anoikis to suppress the dissemination of oncogenically transformed cells. In this manner, resistance to anoikis could contribute to the survival of disseminated tumor cells (DTCs), leading to metastatic colonization.^129^ Therefore, key proteins involved in anoikis could potentially be drug targets for preventing tumor metastasis.^130,131^ Recent studies have demonstrated that ROS may also be involved in anoikis resistance. As reported by Kim’s group, leukotriene B4 receptor-2 (BLT2) can induce anoikis resistance in prostate cancer cells by inducing NOX-mediated ROS accumulation, whereas treatment with DPI attenuates BLT2-promoted anoikis resistance.^132^ Consistently, it has been proved that ROS can activate the Src-mediated ERK and Akt signaling pathways to promote anoikis resistance.^133^ Collectively, these observations have led to the concept that ROS may enable the activation of pro-survival pathways, including the NF-κB and PI3K/AKT pathways, in detached cancer cells, leading to anoikis resistance and malignant transformation.

**Figure 5:**
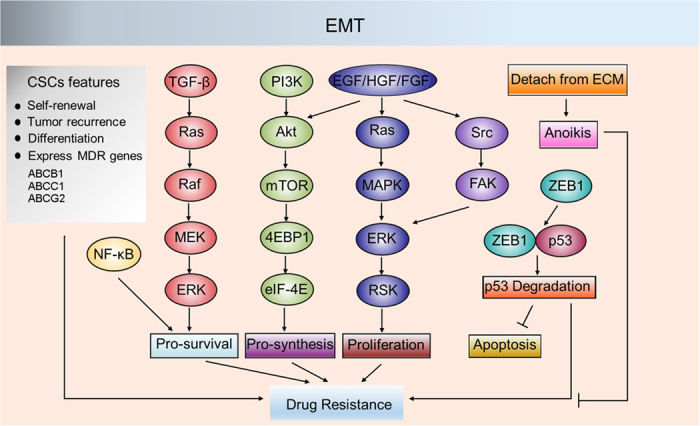
Regulation of drug resistance by EMT. During EMT, multiple pro-survival signaling pathways, such as TGF-β signaling, PI3K/Akt signaling and MAPK signaling, are activated, leading to anoikis resistance and drug resistance in cancer cells. ZEB1 can induce the degradation of p53, enabling cancer cell survival during circulation in the blood. EMT also confers cancer cells with CSC features that allow cancer cells to survive stress induced by chemotherapy and radiotherapy. EMT, epithelial–mesenchymal transition; TGF-β, transforming growth factor β; PI3K, phosphoinositide 3-Kinase; MAPK, mitogen-activated protein kinase; ZEB1, zinc finger E-box binding homeobox 1; CSCs, cancer stem cells; MEK, mitogen-activated protein kinase/ERK kinase; RSK, ribosomal S6 kinase; FAK, focal adhesion kinase; ECM, extracellular matrix; EGF, Epidermal growth factor; HGF, Hepatocyte growth factor; FGF, fibroblast growth factors.

Some hypotheses have been developed regarding the role of EMT in drug resistance. For instance, EMT could confer cancer cells with cancer stem cell (CSC)-like characteristics,^50^ consistent with evidence that either TGF-β treatment or overexpression of the EMT-inducing transcription factors (Snail, Twist) increases the CD44^+^/CD24^−^ subpopulation.^134^ Cellular differentiation markers, such as CD24, CD44 and CD133, are significantly correlated with EMT-associated markers, which are processed by the NF-κB signaling pathway.^135^ Previous studies have suggested that gemcitabine-treated SW1990 gemcitabine-resistant cells show high levels of EMT markers and the CD24^+^CD44^+^/CD133^+^ ratio, implying a link between cell stemness and the EMT phenotype. These changes can be reversed by p65 interference. This result suggests that EMT is an adept mechanism through which cells develop a stem-like phenotype to survive lethal stimuli. Intriguingly, it has been reported that SOD2 could reverse the conversion of CD44^−^ cells to CD44^+^ cells, suggesting that ROS may be involved in the transformation of cancer stem cell-like characteristics.^136^ It is worth noting that a CD44 variant (CD44v) could protect cancer cells against excessive ROS by stabilizing the cystine-glutamate transporter xCT, which contributes to chemotherapy resistance,^137,138^ suggesting that targeting CD44v or xCT may sensitize cancer cells to chemotherapy. CSCs, known as ‘roots’ of aggressive tumors, are believed to be associated with tumorigenesis, tumor recurrence, and tumor metastasis due to their capacity for long-term self-renewal and differentiation into various tumor bulk populations, as well as their resistance to chemotherapy and ionizing radiation.^139^ Because chemotherapeutic agents often target tumor cells that have a rapid proliferation rate, CSCs, which are primarily quiescent and possess an accelerated DNA repair system, could survive stress induced by chemotherapeutic agents. Furthermore, CSCs exhibit high levels of multi-drug resistance (MDR) proteins (such as ABCB1, ABCC1 and ABCG2) to decrease drug influx and promote tumor cell survival.^140^ In addition, pro-survival signaling pathways, such as the TGF-β, NF-κB and PI3K/Akt signaling pathways, are activated during EMT, conferring tumor cells with resistance to chemotherapeutic agent-induced death signals (Figure 5).^141^ Recent studies have shown that CSCs exhibit low levels of ROS to maintain self-renewal, and the advanced antioxidant capacity of CSCs confers resistance to detrimental oxidative stress induced by multiple chemotherapeutic agents (such as cisplatin, doxorubicin and arsenic trioxide).

Cancer cells possess higher levels of ROS compared with those of normal cells due to their rapid metabolic rate.^142^ Continuous low or moderate levels of ROS lead to the activation of several pro-survival signaling pathways. However, high levels of ROS are detrimental due to the induction of DNA damage or the activation of death-associated pathways.^143^ Adaptive antioxidant systems are engaged in cancer cells to counteract toxic ROS. To this end, destroying a fine-tuned antioxidant system with specific inhibitors or inducing excessive levels of ROS beyond the antioxidant capacity of cancer cells could be attractive strategies for cancer therapy.^144^ However, although ROS inducers have been reported to be effective in several cases, drug resistance still seems to occur.^17^ For example, it has been reported that daunomycin/AraC treatment contributes to the upregulation of P-glycoprotein (P-gp) in acute myeloid leukemia (AML) patients. In addition, acute increased expression of MDR1 was observed in patients with unresectable pulmonary sarcoma metastases after isolated single lung perfusion with doxorubicin was performed.^145^ Another study indicated that STAT3 contributes to doxorubicin resistance by upregulating SOD2 and enhancing intracellular antioxidant capacity in ABC-DLBCL cells.^146^

With the aim of developing novel drugs to overcome drug resistance, chemical agents to modulate redox homeostasis are being extensively studied to determine whether they can reverse the EMT process and drug resistance.

### Targeting metal-mediated redox homeostasis as a therapeutic prevention for EMT

Cellular metals (for example, zinc, chromium, CoCl_2_ and nickel compounds) are currently attracting attention because dysregulated metal levels are associated with tumor angiogenesis, tumor cell EMT and tumor cell proliferation due to their ability to modulate ROS.^147^ Here, we will discuss the roles of metal-regulated redox homeostasis in the EMT process of tumor cells.

Zinc is an indispensable component of copper-zinc superoxide dismutase (CuZnSOD) in the cellular antioxidant system.^148^ Moreover, zinc can also reduce ROS production by inhibiting the integration of the cell membrane with iron or copper.^149^ Notably, many critical EMT-inducing transcription factors, such as Twist, Snail and ZEB, are zinc finger proteins.^8^ Increasing evidence has demonstrated that the redox state is critical for regulating Zn^2+^ and its transporters, whereas zinc can in turn modulate cellular ROS levels.^150^ In lung cancer cells, zinc treatment induces EMT by increasing O_2_^•−^ levels, which can be attenuated by MnTBAP, a specific superoxide inhibitor.^151^ Moreover, the zinc transporter ZIP10 (SLC39A10) has been reported to stimulate EMT by inactivating GSK-3β and downregulating E-cadherin.^152^ Another zinc transporter, LIVI, has also been identified as an important regulator of the nuclear translocation of Snail and STAT-mediated EMT.^153^ These observations indicate that zinc and zinc transporters regulate EMT, at least in part, by modulating the intracellular redox state. However, the underlying mechanisms by which zinc regulates cancer cell EMT still needs further investigation.

Hexavalent chromium [Cr^6+^] is widely accepted as a carcinogen and is associated with lung inflammation, kidney damage and nasal ulcers.^154,155,156^ Recent studies have shown that Cr^6+^ can induce EMT and cell invasion to promote oncogenic transformation in lung epithelial cells. This effect is closely linked with ROS accumulation induced by Cr^6+^.^157^ Moreover, it has been reported that Cr^6+^ treatment can also induce the expression of both EMT and stem cell markers in renal epithelial HK-2 cells, which can be restored by the antioxidant vitamin C (Vit C).^158^

CoCl_2_, a hypoxia mimetic agent, can induce ROS production and nuclear localization of HIF-1α.^159^ It has been reported that CoCl_2_ treatment promotes EMT in colorectal cancer cells. Dieckol treatment reversed ROS levels and restored EMT-related morphological changes, suggesting that dieckol is a potential therapeutic for metastatic colorectal cancer.^160^ In line with this finding, another study showed that CoCl_2_ could initiate EMT in breast cancer cells. Tetraethylenepentamine (TEPA), a copper chelator, was capable of inhibiting CoCl_2_-induced ROS accumulation and EMT progression. This study suggested that copper is essential for maintaining cellular antioxidant ability, and copper chelators may be promising drugs for metastatic tumors.^161^

It is well documented that treatment with nickel compounds results in ROS accumulation and DNA damage that promote carcinogenesis.^162^ Recent studies have shown that nickel compounds can promote EMT by ROS generation and induce malignant transformation in lung cancer cells.^163^ Nickel induces ROS accumulation by upregulating NOX1 and SOD2 and downregulating catalase and GPX1/2. It is plausible that nickel-induced gene silencing, especially of E-cadherin, is attributed to ROS generation.^164^ It has been confirmed that tiron (a superoxide anion scavenger) treatment decreases cellular O_2_^•−^ levels and restores NiCl_2_-induced EMT progression. Furthermore, NAC has proven to be one of the most efficient antioxidants in alleviating NiCl_2_-induced EMT.^163^ Recent studies have also revealed that nickel soluble salts can reverse nickel toxicity by upregulating the expression of ABCB1, a multi-drug resistance P-gp.^165^

### Targeting redox homeostasis for EMT prevention

Because ROS play critical roles in EMT progression, it is rational that antioxidants/ROS-inducers or inhibitors/agonists of antioxidant enzymes may hold promise for single or combinational use in cancer therapy.^166^ Antioxidants such as NAC, DPI, vitamin C, ebselen and MPG have been used to retard EMT progression *in vitro.* NAC, one of the most important antioxidants, has been demonstrated to prevent spontaneous metastasis of mouse lung carcinoma cells by repressing mitochondria-derived ROS.^58,167^ Furthermore, a number of natural antioxidant agents have been found to inhibit tumor metastasis. For example, apigenin can inhibit EMT to circumvent tumor migration in human hepatocellular carcinoma (HCC) cells by inactivating the NF-κB/Snail cascades.^168^ In addition, galangin, a flavonoid that shows anticancer properties, can also inhibit cell invasion by restraining EMT in human renal cancer cells.^169^ Unlike apigenin, galangin induces ROS accumulation that leads to cell death. However, the role of ROS in galangin-suppressed EMT needs further investigation. The intracellular antioxidant system is also involved in EMT establishment. For instance, miR-212 has been reported to target manganese superoxide dismutase (MnSOD) to inhibit the invasion and pulmonary metastasis of colorectal cancer cells.^170^ Peroxiredoxin 1 (Prx1), another important antioxidant enzyme, has been demonstrated to affect nicotine-induced EMT in oral squamous cell carcinoma (OSCC). Silencing Prx1 resulted in inactivation of the NF-κB pathway and suppression of the EMT process, suggesting that Prx1 is a potential therapeutic target for cancer cell EMT.^171^ As mentioned above, EMT confers cancer cells with CSC characteristics so that they can escape from chemotherapeutic stress. CSCs exhibit well-developed antioxidant systems to buffer ROS in response to chemoradiotherapy.^172^ For example, overexpression of CD44v maintains low levels of ROS in CSCs by stabilizing xCT and promoting the uptake of cystine, an essential component of GSH synthesis.^173^ Therefore, targeting CD44v or xCT or enhancing cellular ROS levels beyond the threshold of CSCs may be a feasible strategy for cancer therapy.^137,174^ Many conventional chemotherapeutic agents can induce ROS accumulation and result in cancer cell damage due to DNA breakage.^142^ However, ROS accumulation also leads to EMT activation through the upregulation of MMPs, Snail and FoxOs.^175,176^ In addition, ROS can also activate pro-survival signaling pathways to endow cancer cells with anoikis resistance. For example, ROS activate the ligand-independent EGFR and promote the degradation of Bim (a pro-apoptotic protein) by the oxidative activation of Src.^133^ By contrast, diminished glucose oxidation due to the inhibition of pyruvate dehydrogenase (PDH) attenuates oxidative stress, resulting in anoikis resistance and metastasis.^177^ These contradictory results suggest that the role of antioxidants in cancer therapy is complex and needs further elaboration. Recently, it has been reported that a high O_2_^•−^ to H_2_O_2_ ratio is linked with anoikis resistance, and reducing this ratio sensitizes cancer cells to apoptosis. In this regard, NOX4 may emerge as a potential target because it specifically induces H_2_O_2_ production. 4-Me, an amino endoperoxide, can promote cell death in NOX4 overexpressed cancer cells by modulating the activity of NOX4.^178^ NAC or NOX4-targeted siRNA can abolish the pro-apoptosis activity of 4-Me. Given the paradoxical role of ROS in cancer therapy, further studies are urgently needed to uncover the mysterious role of redox regulation in cancer progression, which will help correctly guide the redox-targeting strategy in cancer therapy.

## Conclusions

Tumor metastasis is an ongoing challenge in tumor therapy. Although several signaling pathways (such as the TGF-β, NF-κB, HIF-1α signaling pathways) involved in tumor metastasis have been investigated, new insights are urgently needed to extend our understanding of the intrinsic molecular events that drive tumor cell metastasis.^179^ EMT, a driving force in mediating tumor cell migration, invasion and tumor progression, is responsible for tumor metastasis, as well as drug resistance, and holds the potential to be a drug target for advanced tumor therapy.^180^ Current studies have shown that ROS can influence the function of various key proteins involved in the EMT process through reversible or irreversible oxidative modifications on free cysteine residues. Emerging evidence suggests that ROS accumulation leads to increased cell mobility, diminished cell–cell conjunctions, remodeled cytoskeleton, downregulated epithelial cell markers, upregulated mesenchymal makers and degraded ECM. Buffering ROS using antioxidants, such as NAC and MPG, results in attenuated EMT progression. However, antioxidant treatment may lead to the survival of CSCs and DTCs, implying a dual role of antioxidants in cancer therapy. Interestingly, as mentioned above, different types of ROS seem to have alternative effects on EMT, suggesting that specific inhibitors or inducers of ROS may have different functions. Thus, further studies are required to elucidate these underlying mechanisms. Furthermore, ROS generated from different sources (NOX, 5-LOX, or mitochondria) may contribute to distinct outcomes, further supporting the need to comprehensively understand the regulatory mechanisms underlying ROS. Targeting redox regulation for preventing EMT and tumor metastasis is clearly promising. However, redox-based strategies may play dual roles in cancer therapy as redox modulators themselves may lead to drug resistance. In this regard, combining redox modulators with conventional chemotherapy may benefit therapeutic efficacy.
